# Interaction between oxygen saturation and renal function on 30-day mortality in emergency department patients

**DOI:** 10.1038/s41598-026-45757-x

**Published:** 2026-03-27

**Authors:** Ahmad Zwawi, Per Swärd, Felix Forsberg, Olle Melander, Ulf Ekelund, Anders Björkelund, Per Wändell, Axel C. Carlsson, Toralph Ruge

**Affiliations:** 1 Department of Clinical Sciences Malmö, Department of Internal Medicine, Lund University, Lund, Sweden; 2 Clinical and Molecular Osteoporosis Research Unit, Departments of Orthopedics and Clinical Sciences, Skåne University Hospital, Lund University, Malmö, Sweden; 3 Department of Emergency and Internal Medicine, Skånes University Hospital, Malmö, Sweden; 4 Emergency medicine, Department of Clinical Sciences Lund, Lund University, Lund, Sweden; 5 Department of Emergency Medicine, Skåne University Hospital, Lund, Sweden; 6 Centre for Environmental and Climate Science, Lund University, Lund, Sweden; 7 Department of Neurobiology, Care Sciences and Society, Division of Family Medicine and Primary Care, Karolinska Institutet, Huddinge, Sweden; 8 Center for Primary Health Care Research, Lund University, Malmö, Sweden; 9 Academic Primary Health Care Centre, Region Stockholm, Stockholm, Sweden; 10 School of Health and Social Studies, Dalarna University, Falun, Sweden

**Keywords:** Emergency medicine, Kidney function, Mortality, Oxygen saturation, Diseases, Medical research, Nephrology, Risk factors

## Abstract

Emerging evidence suggests that bidirectional lung–kidney crosstalk may influence outcomes, but this has not been systematically evaluated in unselected emergency department populations. We therefore examined the association between peripheral oxygen saturation (SpO₂), estimated glomerular filtration rate (eGFR), and 30‑day mortality, and tested whether the prognostic association of oxygenation with mortality differs across levels of kidney function (and vice versa), using an SpO₂×eGFR interaction term to model effect modification We analyzed 12,651 adults with complete data on SpO₂, creatinine-derived eGFR, lactate, C-reactive protein (CRP), RETTS triage, and prespecified covariates from the Skåne Emergency Medicine (Skåne 17/18) cohort (2017–2018). We fitted multivariable logistic regression models including SpO₂ and eGFR (Model 1) and then added an SpO₂×eGFR interaction term (Model 2). Nested models were compared using a likelihood-ratio test, and discrimination was compared using AUROC (DeLong test) based on model-predicted probabilities. In a predefined subgroup with arterial blood gases (*n* = 3,068), we evaluated eGFR in relation to PaO₂/FiO₂ (P/F). In the full cohort, SpO₂ and eGFR were significantly correlated. Adding the SpO₂×eGFR interaction term improved model fit versus the main-effects model (LRT ΔDeviance = 15.77, *p* = 7.17 × 10⁻⁵), but discrimination was essentially unchanged (AUROC 0.744 vs. 0.745; ΔAUROC 0.0009; 95% CI − 0.00227 to 0.00046; DeLong *p* = 0.193). In the interaction model, higher SpO₂ and eGFR were associated with lower 30-day mortality (OR 0.81, 95% CI 0.77–0.85; OR 0.85, 95% CI 0.80–0.91), and the interaction term indicated stronger protection when both were higher (OR 0.90, 95% CI 0.86–0.95). Exploratory subgroup analyses suggested the interaction effect was most pronounced among patients presenting with chest pain. SpO₂ and eGFR showed evidence of interaction in relation to 30-day mortality. Although adding the interaction term improved model fit, it did not meaningfully improve discrimination compared to main model (without interaction). This suggests that, in unselected ED populations, measuring and interpreting SpO₂ and eGFR remains clinically useful, whereas explicitly modeling their interaction is unlikely to add substantial predictive benefit.

**Supplementary Information:**

The online version contains supplementary material available at 10.1038/s41598-026-45757-x.

## Introduction

It is well established that reduced oxygenation may directly impair renal perfusion and tubular function, while declining kidney function can, in turn, worsen pulmonary status through fluid overload and metabolic disturbances^1–3^. This bidirectional interplay in lung–kidney has been described in specific populations, such as those with chronic kidney disease, acute respiratory distress syndrome (ARDS), and sepsis^1–3^.

Both impaired oxygenation and renal dysfunction are recognized as strong predictors of adverse outcomes in a variety of acute and chronic illnesses^4–6^. This is in line with prior emergency care studies in which admission eGFR has been evaluated as a practical marker of kidney function and an independent predictor of short-term adverse outcomes^6,7^. However, the prognostic utility of combining SpO₂, PaO₂/FiO₂, and eGFR, especially at the time of presentation, is unclear, whether the combined burden of impaired oxygenation and renal dysfunction confers excess mortality risk beyond the sum of their contributions.

Given these knowledge gaps, we hypothesized that the prognostic association of oxygenation with short-term mortality may differ according to admission creatinine-derived eGFR (and vice versa), consistent with lung–kidney crosstalk^8^. Accordingly, we tested for effect modification between SpO₂ and eGFR by including an SpO₂×eGFR interaction term within multivariable logistic regression models. The rationale is that concurrent abnormalities in oxygenation and admission creatinine-derived eGFR may reflect more severe systemic illness and multi-organ impairment, potentially altering the relationship between either organ marker and mortality risk. Additionally, in a predefined exploratory subgroup with available arterial blood gas data, we examined a cohort-provided PaO₂/FiO₂ (P/F ratio) variable as an alternative oxygenation measure, alongside eGFR^8,9^.

Therefore, the primary aim of this study was to investigate the relationship between oxygenation (SpO₂; and PaO₂/FiO₂ where available), admission creatinine-derived eGFR, and 30-day mortality in adults admitted via the emergency department. In the full cohort, we assessed whether the prognostic association of oxygenation with mortality differed across levels of eGFR, and vice versa, by including an SpO₂×eGFR interaction term in multivariable logistic regression and comparing nested models with and without the interaction. In the arterial blood gas subgroup, we examined whether analogous models using the P/F ratio instead of SpO₂ showed similar patterns.

## Methods

### Cohort description

The study utilized the Skåne Emergency Medicine (SEM) cohort, a register-based dataset comprising all adult (≥ 18 years) patients who visited any of the eight emergency departments in the Skåne region between January 1, 2017, and December 31, 2018^10^.

### Study population

**Fig. 1:**
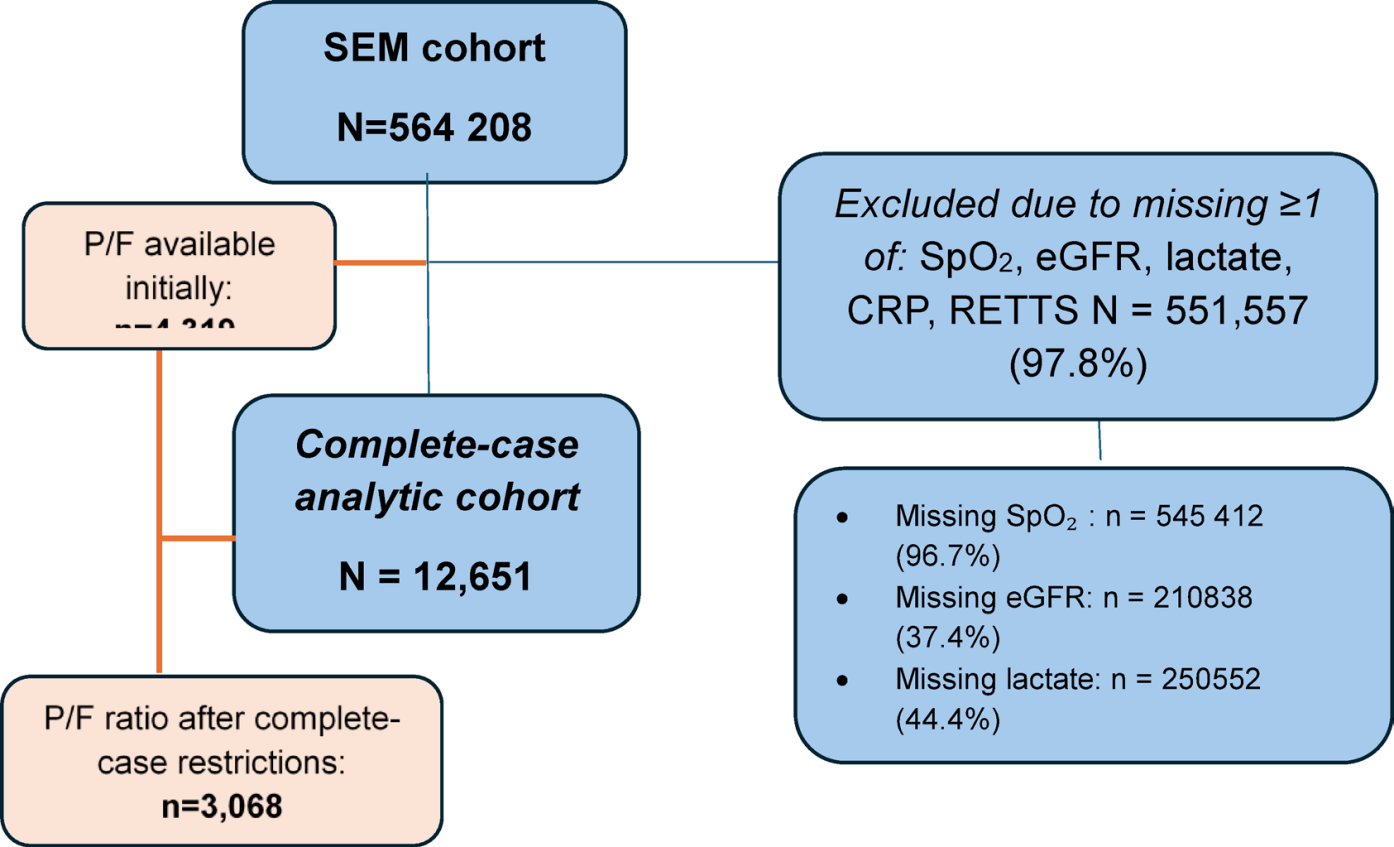
Flow diagram of the study population in the Skåne emergency medicine cohort.

From this comprehensive cohort, we focused on a sample of 12,651 patients after excluding individuals with missing values for any of the following: SpO₂, eGFR, lactate, C-reactive protein (CRP), or RETTS triage scores (Fig. 1)^10^. A subgroup analysis was conducted on available data for PaO_2_/FiO_2_ in 3,068 patients (Fig. 1). The P/F ratio (PaO₂/FiO₂) was available as a precomputed variable in the cohort dataset.

Estimated glomerular filtration rate (eGFR) was calculated from the first serum creatinine measured at emergency department presentation using the Lund–Malmö equation, as recorded in the cohort database. In this study, eGFR was analyzed as a creatinine-derived estimate obtained at presentation rather than as a direct measure of stable baseline kidney function. Peripheral oxygen saturation (SpO₂) was defined as the first recorded SpO₂ value at arrival to the emergency department and reflects the initial clinical assessment at presentation. A single value per patient was used in all analyses. All laboratory measurements, including serum creatinine, C-reactive protein, and lactate, were obtained at presentation to the emergency department as part of routine clinical care. No repeated or time-averaged measurements were used.

### Presenting complaint (RETTS category)

The reason for admission was defined according to the primary presenting complaint category assigned at triage using the Rapid Emergency Triage and Treatment System (RETTS). RETTS is a standardized triage system routinely used in Swedish emergency departments and is based on structured algorithms combining presenting symptoms and vital signs. At triage, each patient is assigned one dominant presenting complaint category, which was used in the analyses. These categories reflect the primary clinical presentation at arrival rather than final diagnosis. Although overlapping clinical features may coexist (e.g., fever in infection), only the primary RETTS category was included for covariable adjustment and exploratory subgroup analyses.

### Statistics

Statistical analyses were conducted in R version 4.2.2. Baseline characteristics between 30-day survivors and non-survivors were compared as follows: continuous variables were assessed for normality and compared using Student’s t-test or the Mann–Whitney U test as appropriate; categorical variables were compared using the chi-square test.

Quantile regression was performed to examine the association between standardized SpO₂ and standardized eGFR at the 25th, 50th, and 75th percentiles. Regression coefficients, standard errors, and p values were reported for each quantile.

The primary outcome was 30-day all-cause mortality. SpO₂ and eGFR were standardized (z-scores; mean = 0, SD = 1).To evaluate whether the association of SpO₂ with mortality differed by the level of admission eGFR (and vice versa), we modeled effect modification by including an SpO₂×eGFR interaction term in multivariable logistic regression. Two prespecified models were fitted and adjusted for age, sex, RETTS (treated as a continuous triage score), lactate (z), CRP (z).

- **Model 1**: main effects of SpO₂ and eGFR.
- **Model 2**: Model 1 plus the SpO₂×eGFR interaction term (product of the two z-scores).

Nested models were compared using a likelihood-ratio test (LRT); Akaike’s Information Criterion (AIC) and Bayesian Information Criterion (BIC) were reported as complementary measures of relative model fit that penalize model complexity (with lower values indicating better fit). Multicollinearity was assessed using variance inflation factors (VIFs; car). Because coefficients in interaction models are conditional, marginal effects were summarized as odds ratios (ORs) for + 1 SD SpO₂ at eGFR − 1/0/+1 SD and for + 1 SD eGFR at SpO₂ −1/0/+1 SD, with 95% confidence intervals.

Discrimination was quantified using the area under the receiver operating characteristic curve (AUROC; pROC) derived from model-predicted probabilities, and AUROCs for Model 1 versus Model 2 were compared using DeLong’s test for correlated ROC curves.

In a predefined subgroup with arterial blood gases, oxygenation was additionally characterized using PaO₂/FiO₂ (P/F ratio), recorded on the kPa scale in the cohort database (1 kPa ≈ 7.5006 mmHg). We fitted analogous multivariable logistic regression models including P/F ratio and eGFR as main effects and tested for effect modification by adding a P/F×eGFR interaction term. Nested models were compared using an LRT, and AUROCs were compared using DeLong’s test based on model-predicted probabilities.

Arterial blood gas data (P/F ratio) were available for 4,319 individuals in the SEM cohort. Of these, 3,068 remained in the analytical ABG subgroup after applying complete-case requirements for the covariates used in the adjusted regression models. Because ABG sampling in the ED is clinically indicated and therefore not random, we compared baseline characteristics between individuals with and without available P/F data to assess potential selection into the ABG subgroup (Supplementary Table 6).

Overall performance (Brier score) and calibration (slope and intercept) were reported. Internal validation used bootstrap resampling (B = 1,000) to obtain optimism-corrected C-index (AUC), calibration slope, and intercept. Analyses used complete-case data. Two-sided *p* < 0.05 was considered statistically significant. Key packages included dplyr, quantreg, stats, rms, emmeans, pROC, car, gtsummary, and gt.

### Missing data and complete-case definition

Primary analyses used a complete-case approach for SpO₂, eGFR, lactate, CRP, and RETTS. Figure 1 shows the study flow, including the numbers of patients excluded because of missing data. Counts for individual missing variables are not mutually exclusive because some patients had missing data for more than one variable. To assess potential selection bias related to missing data, we compared baseline characteristics of included and excluded patients (Supplementary Table 5).

## Results

Among the 12,651 patients included in the analysis, 17% died within 30 days. Table 1 presents the comparison of baseline characteristics between 30-day survivors and non-survivors. Non-survivors were older (*p* < 0.001) and exhibited higher inflammatory and metabolic markers, e.g. higher serum levels of CRP (*p* < 0.001) and lactate (*p* < 0.001) and were more likely to be hospitalized. They also had lower measures of respiratory and renal function: SpO₂ (*p* < 0.001), eGFR (*p* < 0.001), and P/F ratio (*p* < 0.001). In addition, a greater proportion of non-survivors were assigned a “Red” urgency category in the RETTS triage system (*p* < 0.001).

**Table 1:** Background characteristics for 30-day mortality in the Skåne emergency medicine cohort.

Variables	All	Survivor (30-day), *n* = 10 512 (83%)	Non-survivor (30-day) *N* = 2 139 (17%)	*P*-value
Demographic				
Age, years, Median (IQR)	74 (63–82)	73 (61–81)	80 (72–86)	< 0.001
Sex-male	6113 (48.3)	5101 (48.5)	1012 (47.3)	0.371
Biomarkers				
CRP, mg/L, Median (IQR)	21 (5.3–77)	18 (5–69)	41 (10–112)	< 0.001
Lactate, mmol/L, Median (IQR)	1.9 (1.3–3.1)	1.9 (1.3–2.5)	2.6 (1.6–3.7)	< 0.001
eGFR, mL/min/1.73 m, ±SD	59 ± 28	61 ± 28	47 ± 27	< 0.001
SpO₂, (%), Median (IQR)	95 (90–98)	95 (91–98)	94 (88–98)	< 0.001
*P/F ratio (kPa), Median (IQR)	37 (22–53)	39 (23–55)	29 (18–46)	< 0.001
RETTs				
Blue, n (%)	61 (0.5)	58 (0.6)	3 (0.1)	< 0.001
Green, n (%)	119 (0.9)	105 (1)	14 (0.7)	
Yellow, n (%)	2459 (19.4)	2198 (20.9)	261 (12.2)	
Orange, n (%)	4784 (37.8)	4143 (39.4)	641 (30)	
Red, n (%)	5228 (41.3)	4008 (38.1)	1220 (57)	
The reason for admission				
Dyspnea, n (%)	4390 (34.7)	3614 (34.4)	776 (36.3)	0.097
Infection	1125 (8.9)	900 (8.6)	225 (10.5)	0.004
Fever	699 (5.5)	595 (5.7)	104 (4.9)	0.155
Abdominal pain, n (%)	887 (7)	798 (7.5)	98 (4.6)	< 0.001
Chest pain, n (%)	841 (6.6)	755 (7.2)	86 (4)	< 0.001

*P/F ratio was available for 3,068 patients (arterial blood gas subgroup) and is expressed in kPa (1 kPa ≈ 7.5006 mmHg). P-values were tested using the Mann–Whitney U test or two-sided t-test for continuous variables. Categorical variables were tested using the chi-square test. Abbreviations: CRP, C-reactive protein; RETTS, Rapid Emergency Triage and Treatment System; P/F ratio, PaO₂/FiO₂; SD, standard deviation; IQR, interquartile range.

The most common reason for admission was dyspnea (34.4%), followed by infection (8.9%), abdominal pain (7.0%), chest pain (6.6%), and fever (5.5%). Compared to survivors, non-survivors were more likely to present with infection (8.6% vs. 10.5%, *P* = 0.004), and less likely to be admitted with abdominal pain (7.5% vs. 4.6%, *P* < 0.001) or chest pain (7.5% vs. 4.6%, *P* < 0.001).

Of 564,208 eligible individuals in the SEM cohort, 12,651 (2.2%) had complete data for SpO₂, eGFR, lactate, CRP, and RETTS and were included in the primary analyses (Fig. 1). Compared with excluded individuals, included individuals were older, more often hospitalized, had higher 30-day mortality, and had markedly higher RETTS urgency (Supplementary Table 5).

### Quantile regression of standardized SpO_2_ and eGFR

Quantile regression was performed to examine the relationship between standardized arterial SpO₂ and standardized estimated glomerular filtration rate at the 25th, 50th, and 75th percentiles (Table 2).

**Table 2:** Quantile regression in which Z-normalized SpO₂ predicts Z-normalized eGFR in the Skåne emergency medicine cohort.

Quantile	Co-efficient (standard error)	*P*-value
25th	0.031 (0.014)	0.045
50th	0.056 (0.012)	< 0.001
75th	0.083 (0.013)	< 0.001

At the 25th percentile (τ = 0.25), Z sat was positively associated with Z_eGFR (β = 0.031; SE = 0.014; *p* = 0.045), indicating that each 1 SD increase in SpO₂ corresponded to a 0.03SD increase in eGFR in the lower quartile of eGFR. At the median (50th percentile), the association was slightly stronger (β = 0.056; SE = 0.012; *p* < 0.001), and a 1 SD increase in SpO₂ was linked to a 0.056 SD increase in eGFR. The strongest association was observed in the upper quartile (75th percentile) (β = 0.083; SE = 0.013; *p* < 0.001), with each SD increment in SpO₂ predicting a 0.08 SD increased in eGFR.

### Predictive performance of the eGFR–SpO_2_ interaction

To evaluate effect modification between oxygenation and kidney function, we compared a main-effects model (Model 1) with an interaction model (Model 2) that included the SpO₂×eGFR interaction term. Adding the interaction term improved model fit versus the main-effects model (LRT ΔDeviance = 15.77 on 1 df, *p* = 7.17 × 10⁻⁵). However, discrimination was essentially unchanged between Model 1 and Model 2 (AUROC 0.744 vs. 0.745; ΔAUROC 0.0009; 95% CI − 0.00227 to 0.00046; DeLong *p* = 0.193).

### Logistic regression for 30-Day mortality

In multivariable logistic regression (Table 3), parameter estimates from Model 1 and Model 2 are shown. Model comparison results (LRT and AUROC) are reported above. However, discrimination was essentially unchanged between the main-effects and interaction model (AUROC 0.744 vs. 0.745; ΔAUROC 0.0009; 95% CI − 0.00227 to 0.00046; DeLong *p* = 0.193).

**Table 3:** Multivariable logistic regression, for 30-day mortality in the Skåne emergency medicine cohort: main effects vs. main + interaction.

Variables	Model 1: main effects only			Model 2: main effect + interaction		
	OR	95% CI	*p*-value	OR	95% CI	*p*-value
SpO₂ (per SD)	0.85	0.81, 0.89	< 0.001	0.81	0.77, 0.85	< 0.001
eGFR (per SD)	0.86	0.81, 0.92	< 0.001	0.85	0.80, 0.91	< 0.001
Sex (male)	0.94	0.85, 1.04	0.20	0.94	0.85, 1.04	0.20
Age	1.05	1.04, 1.05	< 0.001	1.05	1.04, 1.05	< 0.001
RETTS	1.50	1.40, 1.60	< 0.001	1.50	1.40, 1.61	< 0.001
Lactate (z)	1.51	1.44, 1.58	< 0.001	1.51	1.44, 1.58	< 0.001
CRP (z)	1.19	1.14, 1.24	< 0.001	1.18	1.13, 1.24	< 0.001
SpO₂ (per SD) * eGFR (per SD)	NA	NA	NA	0.90	0.86, 0.95	< 0.001

CI = Confidence Interval, OR = Odds Ratio. Model 1: SpO₂ and eGFR (main effects). Model 2: SpO₂, eGFR, and SpO₂×eGFR. Adjusted for age, sex, RETTS, lactate, and CRP. Continuous predictors standardized (mean = 0, SD = 1). LRT for interaction *p* < 0.001. VIFs all < 1.3. ORs are per + 1 SD; in Model 2, main-effect ORs are conditional on the other predictor at its mean. Discrimination: AUROC(Model 1) = 0.744 and AUROC(Model 2) = 0.745; DeLong *p* = 0.193.

In the main-effects model, higher SpO₂ (OR 0.85, 95% CI 0.81–0.89; *p* < 0.001) and higher admission eGFR (OR 0.87, 95% CI 0.82–0.93; *p* < 0.001) were each associated with lower 30-day mortality (Table 3). In the interaction model, both effects remained protective (SpO₂ OR 0.81, 95% CI 0.77–0.85; *p* < 0.001; eGFR OR 0.86, 95% CI 0.81–0.92; *p* < 0.001) and the interaction was significant (OR 0.90, 95% CI 0.86–0.95; *p* < 0.001), indicating effect modification such that the association of one marker depended on the level of the other (Table 3; Supplementary Tables S1 and S2).

Marginal-effects estimates showed strengthening of one marker’s association at higher levels of the other: SpO₂ per + 1 SD at eGFR − 1/0/+1 SD yielded ORs 0.90/0.81/0.73 (all *p* < 0.001), and eGFR per + 1 SD at SpO₂ −1/0/+1 SD yielded ORs 0.96 (*p* = 0.28), 0.86, and 0.77 (both *p* < 0.001) (Supplementary Tables S3 and S4).

Internal validation with 1,000 bootstrap samples showed negligible optimism (apparent vs. corrected C: 0.748→0.746; interaction model 0.749→0.747). Calibration remained good (slope ≈ 0.99, intercept ≈ − 0.013; Brier ≈ 0.124).

Exploratory admission-reason subgroup analyses suggested heterogeneity in model performance; adding the SpO₂×eGFR interaction term resulted in minimal changes in discrimination across subgroups (Table 4).

**Table 4:** ROC: AUC analysis for 30-day mortality in the Skåne emergency medicine cohort by SpO₂, eGFR, and SpO₂*eGFR divided into subgroups based on the chief complaint at the ED.

Reason of admission	Variables	AUC (95%CI)	*P*-value
Dyspnea, *n* = 4390	eGFR	0.62 (0.60–0.64)	< 0.001
	SpO₂	0.53 (0.50–0.53)	0.029
	SpO₂*eGFR	0.63 (0.61–0.65)	< 0.001
Infection, *n* = 1125	eGFR	0.64 (0.60–0.68)	< 0.001
	SpO₂	0.54 (0.50–0.58)	0.06
	SpO₂*eGFR	0.64 (0.60–0.68)	< 0.001
Chest pain, *n* = 841	eGFR	0.75 (0.71–0.80)	< 0.001
	SpO₂	0.57 (0.51–0.64)	0.033
	SpO₂*eGFR	0.76 (0.71–0.80)	< 0.001
Abdominal pain, *n* = 887	eGFR	0.70 (0.65–0.76)	< 0.001
	SpO₂	0.52 (0.46–0.58)	0.512
	SpO₂*eGFR	0.70 (0.65–0.76)	< 0.001
Fever, *n* = 699	eGFR	0.58 (0.52–0.65)	0.007
	SpO₂	0.59 (0.53–0.65)	0.004
	SpO₂*eGFR	0.59 (0.53–0.65)	0.004

P/F ratio data were available for 4,319/564,208 (0.8%) individuals in the SEM cohort (Fig. 1). Compared with individuals without P/F data, those with P/F available were older, had higher RETTS urgency, and higher hospitalization and 30-day mortality (Supplementary Table 6), consistent with clinically indicated ABG sampling.

#### Arterial blood gas subgroup (P/F ratio)

In the predefined arterial blood gas subgroup (*n* = 3,068), both P/F ratio and eGFR were independently associated with 30-day mortality in the adjusted main-effects model (P/F OR 0.649, 95% CI 0.536–0.780; eGFR OR 0.829, 95% CI 0.735–0.934) (Table 5). However, there was no evidence of effect modification (P/F×eGFR interaction OR 0.965, 95% CI 0.786–1.18; *p* = 0.734), and the nested-model likelihood-ratio test was non-significant (ΔDeviance = 0.116; *p* = 0.733). Discrimination was unchanged when adding the interaction term (AUROC 0.75319 vs. 0.75332; DeLong *p* = 0.599).

**Table 5:** Multivariable logistic regression for 30-day mortality in the arterial blood gas subgroup (*n* = 3068): main-effects model versus interaction model.

Variables	Model 1: main effects only			Model 2: main effect + interaction		
	OR	95% CI	*p*-value	OR	95% CI	*p*-value
PaO₂/FiO₂ (per SD)	0.65	0.54, 0.78	< 0.001	0.64	0.52, 0.78	< 0.001
eGFR (per SD)	0.83	0.74, 0.93	0.002	0.82	0.73, 0.93	0.002
Sex (male)	1.05	0.86, 1.27	0.6	1.05	0.86, 1.27	0.6
Age	1.04	1.03, 1.05	< 0.001	1.04	1.03, 1.05	< 0.001
RETTS	1.50	1.29, 1.74	< 0.001	1.50	1.29, 1.74	< 0.001
Lactate (z)	1.46	1.37, 1.56	< 0.001	1.46	1.37, 1.56	< 0.001
CRP (z)	1.05	0.97, 1.14	0.2	1.05	0.97, 1.14	0.2
PaO₂/FiO₂ (per SD) * eGFR (per SD)	NA	NA	NA	0.97	0.79, 1.18	0.7

CI = Confidence Interval, OR = Odds Ratio. Model 1: PaO₂/FiO₂ and eGFR (main effects). Model 2: PaO₂/FiO₂, eGFR, and PaO₂/FiO₂ ×eGFR. Adjusted for age, sex, RETTS, lactate, and CRP. Continuous predictors standardized (mean = 0, SD = 1). LRT for interaction *p* = 0.733. VIFs all < 1.3. ORs are per + 1 SD; in Model 2, main-effect ORs are conditional on the other predictor at its mean. Discrimination: AUROC(Model 1) = 0.75319 and AUROC(Model 2) = 0.75332; DeLong *p* = 0.599.

Exploratory supplementary AUC comparisons showed that eGFR alone discriminated 30-day mortality better than SpO₂ (AUC 0.647 vs. 0.546) and better than P/F ratio in the ABG subgroup (AUC 0.650 vs. 0.609) (Supplementary Tables 7–8).

## Discussion

The primary aim of this study was to examine the relationship between oxygenation, kidney function, and 30‑day mortality in adults admitted to the emergency department. In the full cohort, both lower SpO₂ and lower admission eGFR were independently associated with higher short-term mortality. We observed statistical interaction between oxygenation and renal function, but adding the interaction term only modestly improved model fit and did not meaningfully change discrimination, suggesting limited incremental value for risk stratification at the ED population level. How this interaction behaves within specific diagnostic groups or disease entities remains however uncertain.

The interplay between lung and kidney in acute and chronic diseases is supported by clinical and experimental studies^1–3^. Acute hypoxemia can impair renal oxygen delivery, triggering vasoconstriction, oxidative stress, and inflammation, which may precipitate acute kidney injury^4,11–13^. Conversely, renal dysfunction can promote fluid overload and metabolic derangements, contributing to pulmonary oedema and impaired gas exchange^1–3^. Systemic inflammation further amplifies this interplay. Pro-inflammatory cytokines and reactive oxygen species propagate injury across organs, while hypoxemia and reduced eGFR both perpetuate this cycle^1–3,11–13^. In acute respiratory distress syndrome (ARDS) and other acute respiratory conditions, the incidence of AKI is higher than in other critically ill populations, and this is associated with increased mortality^1–3,14^. Similarly, renal fibrosis and chronic kidney disease (CKD) can drive pulmonary complications through persistent inflammation, endothelial dysfunction, and capillary leak^4,13,15,16^. Likewise, in chronic lung disease, hypercapnia and hypoxemia are common, and synergistically, they can cause vasoconstriction, contribute to renal ischemia, damage the renal endothelium, and disturb sodium and water balance^4^.

Pulmonary and renal dysfunction are well-established predictors of poor outcomes across a range of acute and chronic diseases^1,2,4^. In the community-based cohort (*n* ≈ 5,100; adults without COPD), each 1-percentage-point lower SpO₂ was linked to ~ 4% higher all-cause mortality risk^17^ while the PaO₂/FiO₂ ratio was shown to add some predictive accuracy in ARDS and pulmonary embolism cases for hospital mortality^8,9^. Similarly, reduced eGFR on admission correlates with 30-day mortality, with a 15% increased risk per 10 ml/min/1.73 m² decline and almost 4-fold risk at < 15 ml/min/1.73 m² in hospitalized patients^6^. Also, in a community-based cohort, nocturnal hypoxia (defined as SpO₂ < 90% ) was associated with nearly a 3-fold increased risk of accelerated eGFR loss^18^ The high prevalence of lung dysfunction in CKD further underscores the concept of bidirectional organ crosstalk^12,14,19,20^. The kidney’s unique hemodynamics and high oxygen consumption render it particularly susceptible to changes in systemic oxygen delivery.

The modest improvement in predictive accuracy by combining kidney and lung measures, as well as the robust performance of eGFR in patients with chest pain, highlights the clinical importance of admission creatinine-derived eGFR as a prognostic marker in acutely ill populations. This interpretation is also supported by prior studies in emergency care settings, where admission eGFR has shown independent associations with short-term adverse outcomes and mortality^6,7^. Renal‑cardiac interaction during pathogenesis and progression is well‑recognized^20^. At the same time, our marginal‑effects analyses indicate that the prognostic contribution of eGFR is attenuated when SpO2 is low, whereas eGFR becomes a stronger predictor at higher SpO₂ levels (Supplementary Tables S3–S4), suggesting that severe hypoxaemia may overshadow the prognostic information carried by admission eGFR alone. This pattern is compatible with a scenario in which advanced respiratory distress dominates short‑term risk, while preserved oxygenation allows admission eGFR to reflect a mixture of chronic kidney dysfunction, comorbidity burden, and acute physiological disturbance at presentation. In patients presenting with chest pain, the particularly strong discriminative performance of eGFR (Table 4) may reflect established cardio‑renal mechanisms, where reduced renal function both mirrors long‑standing cardiovascular disease burden and exacerbates cardiac stress through volume overload, hypertension, and metabolic derangements^20–22^. Together, these findings imply that admission eGFR should be interpreted in relation to both presenting symptom and to the degree of respiratory impairment, rather than as an isolated risk marker.”

The relative importance of the SpO₂–eGFR interaction is also likely to vary across clinical contexts. In settings with pronounced primary respiratory failure, such as acute respiratory distress syndrome or severe pneumonia, several studies suggest that oxygenation indices and lung injury scores dominate short‑term prognosis, while kidney dysfunction mainly emerges as a downstream complication that further amplifies risk^1–3,7,13^. In contrast, in more heterogeneous emergency populations, chronic comorbidities and pre‑existing renal impairment are common, and our findings indicate that the interaction between oxygenation and admission eGFR adds only limited incremental discrimination beyond the main effects. In the arterial blood gas subgroup, for example, PF ratio and eGFR remained independently prognostic, but the PF, eGFR interaction term did not enhance model performance.

### Clinical relevance

Both lower SpO₂ and lower eGFR were independently associated with higher 30-day all-cause mortality, and both measures therefore provide clinically relevant prognostic information in the emergency department. Although we observed statistical interaction between oxygenation and kidney function in the full cohort, adding the interaction term improved model fit but did not meaningfully improve discrimination. In exploratory supplementary single-predictor AUC analyses, eGFR showed higher discrimination than SpO₂ (and than P/F ratio in the ABG subgroup), but this should not be interpreted as implying that oxygenation is not prognostically important. Rather, our findings support joint bedside interpretation of oxygenation and admission creatinine-derived eGFR, without implementing a separate interaction-based risk index in unselected ED populations.

### Strength and limitations

This study has several strengths, including a large, diverse cohort and robust statistical power. The use of routine clinical measurements (SpO₂, eGFR, P/F ratio) enhances generalizability. However, several limitations merit consideration. As an observational study, causality cannot be inferred. A key limitation of this study is the use of a complete-case analytical design. A substantial proportion of emergency department visits were excluded due to missing data on eGFR, SpO₂, or other covariates, resulting in a reduced and selected analytical cohort. Additionally, the final study population may not be representative of all emergency department attendances, which limits the external validity of the findings. Results should therefore be interpreted with caution. We also lacked reliable information on whether patients were receiving supplemental oxygen at the time of the first ED SpO₂ measurement, which may have introduced exposure misclassification and attenuated the observed association between SpO₂ and 30-day mortality. An additional limitation is that eGFR was derived from a single serum creatinine measurement obtained at emergency department presentation. In acutely ill patients, serum creatinine may be influenced by dynamic changes in kidney function, haemodynamics, muscle metabolism, and analytical variability. Therefore, the calculated eGFR should be interpreted as a creatinine-derived prognostic marker at presentation rather than as a direct measure of stable baseline kidney function or renal functional reserve.

While SpO₂ and P/F ratio are established markers of acute respiratory impairment, in our cohort, these measures were less predictive of 30-day mortality compared to eGFR. Several factors may explain this finding. First, SpO₂ readings can be affected by technical and physiological variables such as skin pigmentation, level of partial pressure, and poor perfusion^23^. Additionally, the PaO₂/FiO₂ ratio requires arterial blood gas analysis, which was not available for all patients, possibly introducing selection bias and reducing statistical power.

Our study population also included a broad spectrum of acutely ill patients, many of whom did not have primary respiratory failure. In such a population, chronic comorbidities, frailty, and acute physiological disturbance at presentation may play a more decisive role in short-term outcomes, and admission eGFR may partly capture this broader risk profile. This is consistent with previous studies reporting that oxygenation measurement through P/F ratio, while highly predictive in patients with severe respiratory disease or ARDS, has less prognostic value as a clinical tool in unselected patients^8^. Furthermore, oxygenation parameters can change rapidly during acute illness, and admission eGFR may reflect a mixture of chronic kidney dysfunction, comorbidity burden, and acute physiological disturbance at presentation. Collectively, these factors may have contributed to the observed predominance of eGFR in mortality prediction in our cohort.

## Conclusion

In this large emergency department cohort, both reduced SpO₂ and lower admission eGFR were associated with increased 30‑day mortality, and we observed evidence of effect modification between these measures, as adding an SpO₂×eGFR interaction term improved model fit compared with a main-effects model.

However, adding an interaction term improved model fit only modestly and did not meaningfully enhance overall discrimination. Admission eGFR showed stronger prognostic discrimination than oxygenation measures in this cohort, supporting eGFR as a key, readily available marker of short‑term mortality risk in this setting, while the clinical utility of explicitly modelling the interaction between oxygenation and kidney function in unselected ED populations remains uncertain.

## Supplementary Information

Below is the link to the electronic supplementary material.

Supplementary Material 1

## Data Availability

Upon reasonable request, all data is available from the first author AZ.

## Abbreviations

AIC: Akaike information criterion
BIC: Bayesian information criterion
ARDS: Acute respiratory distress syndrome
AUC: Area under curve
AUROC: Area under the receiver operating characteristic curve
CI: Confidence interval
CKD: Chronic kidney disease
COPD: Chronic obstructive pulmonary disease
CRP: C-reactive protein
eGFR: Estimated glomerular filtration rate
F: Fraction of inspired oxygen
FiO_2_: Fraction of inspired oxygen
IQR: Inter-quartile range
LRT: Likelihood-ratio test
OR: Odds ratio
P: Partial arterial oxygen pressure
PaO_2_: Arterial oxygen partial pressure (in kPa unit)
P/F: Ratio of partial arterial oxygen pressure (PaO2) to fractional inspired oxygen (FiO_2_) (in kPa unit)
ROC: Receiver operating characteristic
RETTS: Rapid emergency triage and treatment system
Saturation: SpO_2_
SD: Standard deviation
SEM: Skåne emergency medicine cohort
