# Supplementary material for: Interaction between oxygen saturation and renal function on 30-day mortality in emergency department patients
Source: Sci Rep. 2026 Mar 27;16:10518. doi: 10.1038/s41598-026-45757-x (PMC13036071; doi:10.1038/s41598-026-45757-x)
Supplement: Supplementary file 1 — Supplementary Material 1 [file 41598_2026_45757_MOESM1_ESM.docx]

Supplementary Tables

**Supplementary Table S1. Log-regression analysis in which Z-normalized (eGFR *Saturation), Saturation, and eGFR predict 30-day mortality.**

| Models | OR (95% CI) | P-value |
| --- | --- | --- |
| **Model (a)-univariate** |  |  |
| Z-(eGFR*Sat) | 0.90 (0.86-0.94) | <0.001 |
| **Model (b): Model (a) + age+ Sex + RETTs** |  |  |
| Z-(eGFR*Sat) | 0.89 (0.85, 0.94) | <0.001 |
| **Model (c): Model (b) +Z-CRP+Z-Lactate** |  |  |
| Z-(eGFR*Sat) | 0.90 (0.86, 0.95) | <0.001 |
| **Model (d): Model c + reasons of admission.** |  |  |
| Z-(eGFR*Sat) | 0.90 (0.85, 0.95) | <0.001 |

**Supplementary Table S2. Log-regression analysis in which Saturation, and eGFR predict 30-day mortality.**

| Models | OR (95% CI) | P-value |
| --- | --- | --- |
| **Model (a)-univariate** |  |  |
| Z-eGFR | 0.59 (0.56,0.62) | <0.001 |
| Z-SpO2 | 0.84 (0.81,0.88) | <0.001 |
| **Model (b): Model (a) + age+ Sex + RETTs** |  |  |
| Z-eGFR | 0.73 (0.69, 0.78) | <0.001 |
| Z-SpO2 | 0.85 (0.81, 0.89) | <0.001 |
| **Model (c): Model (b) +Z-CRP+Z-Lactate** |  |  |
| Z-eGFR | 0.85 (0.81, 0.89) | <0.001 |
| Z-SpO2 | 0.90 (0.81, 0.92) | <0.001 |
| **Model (d): Model c + reasons of admission.** |  |  |
| Z-eGFR | 0.87 (0.83,0.94) | <0.001 |
| Z-SpO2 | 0.85 (0.81,0.93) | <0.001 |

**Supplementary Table S3. Marginal effect: OR per +1 SD SpO₂ at eGFR −1/0/+1 SD**

| Effect | eGFR level (SD) | OR | LCL | UCL | p |
| --- | --- | --- | --- | --- | --- |
| Per +1 SD SpO₂ | -1 | 0.90 | 0.85 | 0.95 | <0.001 |
| Per +1 SD SpO₂ | 0 | 0.81 | 0.77 | 0.85 | <0.001 |
| Per +1 SD SpO₂ | 1 | 0.73 | 0.67 | 0.79 | <0.001 |

**Supplementary Table S4. Marginal effect: OR per +1 SD eGFR at SpO₂ −1/0/+1 SD**

| Effect | SpO₂ level (SD) | OR | LCL | UCL | p |
| --- | --- | --- | --- | --- | --- |
| Per +1 SD eGFR | -1 | 0.96 | 0.88 | 1.04 | 0.28 |
| Per +1 SD eGFR | 0 | 0.86 | 0.81 | 0.92 | <0.001 |
| Per +1 SD eGFR | 1 | 0.77 | 0.71 | 0.84 | <0.001 |

**Supplementary Table S5. Baseline characteristics of included (complete-case) versus excluded patients in the Skåne Emergency Medicine cohort**

| Variables | Excluded | Included | P-value |
| --- | --- | --- | --- |
| N (%) | 551,557 (97.8) | 12,651 (2.2) | NA |
| Age, years, median (IQR) | 57 (37-74) | 74 (63-82) | <0.001 |
| Sex, male, n (%) | 283318 (51.4) | 6113 (48.3) | <0.001 |
| Hospitalization, n (%) | 139374 (25.3) | 10722 (84.4) | <0.001 |
| 30-day mortality | 11232 (2) | 2139 (16.9) | <0.001 |
| RETT-s triage |  |  | <0.001 |
| Blue, n (%) | 36244 (7.1) | 61 (0.5) |  |
| Green, n (%) | 38238 (7.5) | 119 (0.9) |  |
| Yellow, n (%) | 284311 (55.7) | 2459 (19.4) |  |
| Orange, n (%) | 121580 (23.8) | 4784 (37.8) |  |
| Red, n (%) | 30360 (5.9) | 5228 (41.3) |  |

Given the very large sample size, small absolute differences may yield statistically significant p-values; results should be interpreted in terms of magnitude.

**Supplementary Table S6. Baseline characteristics of patients with versus without available PaO₂/FiO₂ (P/F) ratio in the Skåne Emergency Medicine cohort.**

| Variables | Missing (P/F ratio) | Available (P/F ratio) | P-value |
| --- | --- | --- | --- |
| N (%) | 559,889 (99.2) | 4,319 (0.8) | NA |
| Age, years, median (IQR) | 58 (37-75) | 68 (53-77) | <0.001 |
| Sex, male, n (%) | 287 581 (51.4) | 1850 (42.8) | <0.001 |
| Hospitalization, n (%) | 146123 (26.1) | 3973 (92) | <0.001 |
| 30-day mortality | 12506 (2.2) | 865 (20) | <0.001 |
| RETT-s triage |  |  | <0.001 |
| Blue, n (%) | 36295 (7.0) | 10 (0.2) |  |
| Green, n (%) | 38335 (7.4) | 22 (0.5) |  |
| Yellow, n (%) | 286262 (55.1) | 508 (11.8) |  |
| Orange, n (%) | 125248 (24.1) | 1116 (25.9) |  |
| Red, n (%) | 32942 (6.3) | 2646 (61.5) |  |

Given the very large sample size, small absolute differences may yield statistically significant p-values; results should be interpreted in terms of magnitude.

**Supplementary Table S7. Delong’s test for 30-day mortality in the Skåne Emergency Medicine cohort.**

| Variables | AUC | 95%CI differences of AUCs | P-value |
| --- | --- | --- | --- |
| eGFR * SpO₂ | 0.655 | 0.008 (0.004 to 0.011) | <0.001 |
| eGFR | 0.647 |  |  |
|  | | | |
| eGFR * SpO₂ | 0.671 | 0.109 (0.092 to 0.127) | <0.001 |
| SpO₂ | 0.546 |  |  |
|  | | | |
| Model 1: age + sex + RETTs | 0.697 | -0.015 ( -0.026 to -0.016) | <0.001 |
| Model 2: Model 1+ eGFR * SpO₂ | 0.712 |  |  |
|  |  |  |  |
| Model 1: age + sex + RETTs | 0.697 | -0.012 (-0.016 to -0.006) | <0.001 |
| Model 2: Model 1+ eGFR | 0.709 |  |  |

**Supplementary Table S8. AUC comparison for 30-day mortality in the Skåne Emergency Medicine cohort.**

| **Variables** | **AUC** | **95%CI differences of AUCs** | **P-value** |
| --- | --- | --- | --- |
| **eGFR-PF** | **0.672** | **0.022 (0.011 to 0.033)** | **<0.001** |
| **eGFR** | **0.650** |  |  |
|  | | | |
| **eGFR-PF** | **0.672** | **0.063 (0.038 to 0.088)** | **<0.001** |
| **P/F ratio** | **0.609** |  |  |
|  | | | |
| **Model 1: age + sex + RETTs** | **0.684** | **-0.027 (- 0.038 to -0.016)** | **<0.001** |
| **Model 2: Model 1+ eGFR-PF** | **0.710** |  |  |
|  |  |  |  |
| **Model 1: age + sex + RETTs** | **0.684** | **-0.019 (-0.03 to -0.007)** | **0.001** |
| **Model 2: Model 1+ eGFR** | **0.702** |  |  |
